# Zinc-Doped Bioactive Glass/Polycaprolactone Hybrid Scaffolds Manufactured by Direct and Indirect 3D Printing Methods for Bone Regeneration

**DOI:** 10.3390/cells12131759

**Published:** 2023-06-30

**Authors:** Nafise Elahpour, Isabella Niesner, Cédric Bossard, Nora Abdellaoui, Valérie Montouillout, Franck Fayon, Christine Taviot-Guého, Tina Frankenbach, Alexander Crispin, Pardis Khosravani, Boris Michael Holzapfel, Edouard Jallot, Susanne Mayer-Wagner, Jonathan Lao

**Affiliations:** 1 Laboratoire de Physique de Clermont (LPC), Université Clermont Auvergne, CNRS/IN2P3, F-63000 Clermont-Ferrand, France; nafise.elahpour@clermont.in2p3.fr (N.E.);; 2 Department of Orthopaedics and Trauma Surgery, Musculoskeletal University Center Munich (MUM), LMU University Hospital, LMU Munich, 80336 Munich, Germany; 3 Conditions Extrêmes et Matériaux: Haute Température et Irradiation (CEMHTI), CNRS-UPR3079, Université Orléans, F-45071 Orléans, France; 4 Institut de Chimie de Clermont-Ferrand, Université Clermont Auvergne, CNRS/UMR 6296, F-63000 Clermont-Ferrand, France; 5 Institute for Medical Information Processing, Biometry, and Epidemiology (IBE), Ludwig-Maximilians-Universität München, 81377 Munich, Germany; 6 Flow Cytometry Core Facility, Biomedical Center, Ludwig-Maximilians-Universität München, 82152 Planegg, Germany

**Keywords:** sol–gel, bioactive glass, organic-inorganic hybrid, additive manufacturing, human mesenchymal stem cells, bone tissue engineering

## Abstract

A novel organic–inorganic hybrid, based on SiO_2_-CaO-ZnO bioactive glass (BG) and polycaprolactone (PCL), associating the highly bioactive and versatile bioactive glass with clinically established PCL was examined. The BG–PCL hybrid is obtained by acid-catalyzed silica sol–gel process inside PCL solution either by direct or indirect printing. Apatite-formation tests in simulated body fluid (SBF) confirm the ion release along with the hybrid’s bone-like apatite forming. Kinetics differ significantly between directly and indirectly printed scaffolds, the former requiring longer periods to degrade, while the latter demonstrates faster calcium phosphate (CaP) formation. Remarkably, Zn diffusion and accumulation are observed at the surface within the newly formed active CaP layer. Zn release is found to be dependent on printing method and immersion medium. Investigation of BG at the atomic scale reveals the ambivalent role of Zn, capable of acting both as a network modifier and as a network former linking the BG silicate network. In addition, hMSCs viability assay proves no cytotoxicity of the Zn hybrid. LIVE/DEAD staining demonstrated excellent cell viability and proliferation for over seven weeks. Overall, this hybrid material either non-doped or doped with a metal trace element is a promising candidate to be translated to clinical applications for bone regeneration.

## 1. Introduction

Critical-size bone defects cannot heal without additional surgical treatment. They pose a major orthopedic challenge in healthcare, with high costs and patient morbidity [1]. With over two million bone grafting surgeries performed yearly [2], bone grafting remains the second-most common tissue transplantation after blood transfusion. Autografts are still considered the “gold standard” for treatment; however, they suffer from several inherent limitations, including limited availability and increased morbidity due to the need for a surgical donor site. Biobanked allografts as an alternative require costly tissue harvesting and storage procedures and carry an additional risk of infection all while demonstrating only limited osteoinductive and mechanical properties. Therefore, synthetic substitutes are increasingly moving into the focus of research groups [3,4]. Numerous requirements are placed on ideal bone substitute materials in bone tissue engineering (BTE). In addition to good biocompatibility and adequate mechanical properties, the scaffolds should have high osteoinductivity, osteoconductivity, and biodegradability to be colonized with cells and function as suitable tissue templates for bone formation [5].

In line with these requirements, bioactive glasses (BG) are of the highest interest among synthetic bone substitutes. Once implanted, they trigger physicochemical reactions with body fluids and subsequent delivery of osteostimulating degradation products (e.g., Si, Ca, P ions) capable of regulating and even amplifying in vivo osteogenesis: for instance, among BG-leaching products, Si(OH)_4_ orthosilicic acid species are known to stimulate osteoblast proliferation and differentiation [6]. This interaction between the implant and host finally results in a robust interfacial bioactive layer that will be arduous to be explanted even when applying force, due to the strong BG bonding with bone.

Beyond bone/cartilage regeneration, recent research discloses promising results in wound healing and soft tissue repair [7]. Indeed, BG consists of a relatively easily tunable glassy matrix, predominantly when synthesized through the sol–gel route, which allows the incorporation of a wide variety of “therapeutic” ions that can trigger targeted biological assets once delivered in the body. Those desirable assets include osteogenesis (e.g., Sr^2+^ [8,9,10], Li^+^ [11]), angiogenesis (Co^2+^ [12], Cu^2+^ [13,14], Zn^2+^ [15]), as well as immune-modulatory (BO_3_^3−^, Cu^2+^, Zn^2+^ [16]), anti-inflammatory (Zn^2+^ [17]), and anti-bacterial activity (Ag^+^ [18], Cu^2+^ [16], Zn^2+^ [19]). As can be seen, Zn ions are desirable due to their simultaneous actions on multiple levels. In fact, zinc as the sixth-most abundant trace element in the human body plays a crucial role in bone homeostasis, turnover, mineralization, and ECM (extracellular matrix) synthesis [20]. Bone tissue is the major reservoir of Zn in human body [21,22]. Studies suggest that Zn supplementation upregulates osteoprotegrin (OPG) expression. Despite insufficient knowledge about cellular/molecular pathways through which zinc promotes bone growth, it is known that it can favorably affect osteoblast and chondrocyte functions while prohibiting osteoclastic bone resorption [21,23]. It is noteworthy that all Zn amount in the blood is bound with albumin, α2-macroglobulin which increases the apparent binding of Zn^2+^ and diminishes free Zn^2+^ concentration in the serum to less than 1 nM [24]. From a materials point of view, a first challenge is thus to be able to deliver significant amounts of Zn from the glass to the medium. Zn in the glass is present in the form of zinc oxide (ZnO), which partially dissolves in aqueous media to yield Zn^2+^ cations, which, either in a free or complex form, significantly contribute to the biological effect, as demonstrated for the antibacterial activity in literature [25]. A second challenge is related to the bone-like apatite-forming ability of BG—usually referred to as in vitro bioactivity, Zn being known to impede the transformation of amorphous calcium phosphate (CaP) to crystalline hydroxycarbonate apatite (HCA) [26]. The higher the Zn^2+^ content, the slower the HCA deposition; likewise with the rate of nucleation and later precipitation [27]. In addition, pH adjustment, media circulation/refreshment, the porosity of the material, and incubation duration can obviously influence the HCA precipitation rates [28,29]. 

Here, our strategy to tackle these two challenges was to first act on the porosity of the BG material with the aid of additive manufacturing. Considering the brittleness of raw BG scaffolds, we associate the inorganic BG phase with a challenging but “3D-printing-friendly” polymeric phase, namely polycaprolactone (PCL), which is a polymer of choice in the bioprinting field due to its adequate melting point as well as rheological and shear-thinning properties [30]. We investigate the impact of Zn incorporation inside SiO_2_-CaO BG/PCL hybrid scaffolds that previously showed remarkable performance supporting bone growth in a challenging critical-mice calvaria model [31]. Hybrids consisting of 30 wt% BG based on SiO_2_-CaO-ZnO (75/15/10 Si/Ca/Zn relative atomic percent) and 70 wt% PCL were produced by conducting the silica sol–gel process inside a solution of PCL [32]. SiO_2_-CaO-ZnO BG/PCL hybrid scaffolds were additively manufactured by FDM-based (fused deposition modeling) techniques involving direct and indirect 3D printing. We hypothesized that the two completely different porous structures obtained could be used to modulate the Zn delivery and thus have an inhibitory effect on Hydroxyapatite (Hap) formation and subsequent biological effects. Similarly, the degradation of the materials, ion delivery and Hap-formation ability were investigated in two different media, a protein-free medium (standard saline SBF solution) and a protein-based solution (Mueller–Hinton broth medium), hypothesizing that the ability of proteins to create soluble complexes with metallic ions could impact the Zn release and in vitro bioactivity. Along with a complete set of physicochemical characterization, the in vitro cell behavior was evaluated through direct and indirect cell viability assays involving bone-marrow-derived human mesenchymal cells (hMSCs) to evaluate the immense potential of hybrid scaffolds for BTE applications.

## 2. Materials and Methods

### 2.1. Synthesis

The sol–gel synthesis of bioactive glasses proceeded according to our conventional protocol detailed elsewhere [32]. Glasses with relative atomic percentage ratios of 75/25 Si/Zn, 75/15/10 Si/Ca/Zn (relative atom%) were synthesized. The binary system was used as a control group to evaluate the influence of Zn incorporation. Analytical-grade reagents comprising of Tetraethylorthosilicate (TEOS) (99% purity, Sigma-Aldrich, Saint-Quentin-Fallavier, France) and zinc methoxide (99.9% purity, Aldrich^®^, Saint-Quentin-Fallavier, France) were used as the sol–gel precursors for Si and Zn. The calcium source was obtained by overnight calcination of a CaCO3 powder (Normapur, VWR^®^, Fontenay-sous-bois, France) at a heating rate of 1°/min up to 1000 °C plateaued for 1 h. After one hour of cooling down under vacuum, obtained CaO powder was freshly used. 

The sol–gel process was started by hydrolyzing TEOS in absolute ethanol with the addition of 2 M HCl (diluted from 37% fuming HCl, Sigma-Aldrich) for 30 min (molar ratio of ethanol: H_2_O:TEOS:HCl = 3.7:2:1:0.07). This was pursued by calcium oxide and/or zinc methoxide addition, and then the same amount of ethanol as above was used to aid dissolution of the powder while stirring the mixture (250 rpm) to form the glass network.

Shortly before the gelation of the bioactive glass solution, the sol was divided into two parts. Half of it was kept for 72 h of aging in the same sealed flask and consecutive room temperature (RT) drying for further analyses (X-ray Diffraction (XRD), Nuclear magnetic resonance spectroscopy (NMR), Pair distribution function analysis (PDF)). The obtained xerogel were ground into very fine powder by an agate mortar and pestle prior to analysis.

The other half of the BG sol was used to produce hybrids by mixing it with a solution of (18.2 *w*/*v* %) PCL in tetrahydrofuran (99.9% purity, Sigma-Aldrich) with a 3:7 (*wt*/*wt*) BG sol/PCL solution. The PCL average molecular number varied dependent of the printing method (Mn = 80 kDa or 45 kDa, Sigma-Aldrich). The highly viscous BG/PCL sols were rigorously blended manually, sonicated for 15 min (10 kW, 40 kHz), then stirred in sealed flask for 1 h for homogenization and further condensation. The homogeneous obtained hybrid sol was then processed into scaffolds, but in two different manners based on direct and indirect 3D printing as explained in the following sections.

### 2.2. Additive Manufacturing of Hybrids

#### 2.2.1. Three-Dimensional Direct Printing

The synthesized hybrid mentioned in previous section was left under the laminar flow hood to be dried to form a xerogel. The latter was crushed with a knife mill (IKA A11 basic analytical mill) followed by rinsing in absolute ethanol and drying at RT. The obtained powder was loaded in a metal syringe equipped with a 500-µm nozzle and heated for approximately 1 h (temperatures mentioned below). The syringe was mounted on an FDM bioprinter (Bioscaffolder GeSim mbH), which allows the extrusion of highly viscous materials thanks to a piston-based extrusion conducted at 82 °C. The cartridge and nozzle temperatures were chosen to be 75 °C and 82 °C, respectively; the feed rate and printing speed were equal to 1 and 0.8 mm/s, respectively. The material flow experiencing a change in the section from a few cm diameter (syringe cylinder) to a few mm (the conical attached tip) and finally through the micrometric nozzle made it essential to increase the temperature of the nozzle a few degrees above the temperature of the syringe. Multiple 3-layered scaffolds (radius = 10 mm, infill distance = 600 µm) were prepared.

#### 2.2.2. Three-Dimensional Indirect Printing

**Figure 1:**
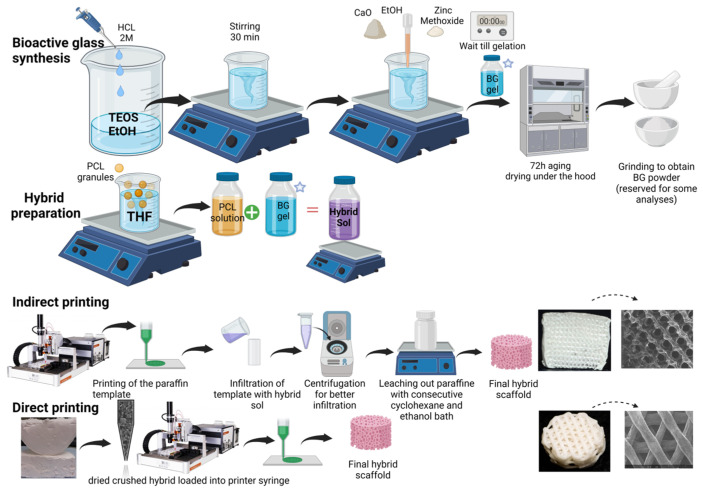
Schematic representation of synthesis and additive manufacturing of hybrid scaffolds, created with BioRender.com, accessed on 21 June 2023.

Based on a template leaching method, paraffin template molds were printed from paraffin granules loaded in a metal syringe equipped with a 300 µm nozzle. The feed rate, speed, cartridge, and nozzle temperature were set to 1 mm/s, 1.8 mm/s, 46 °C, and 47 °C, respectively. The paraffin sacrificing templates were geometrically defined as circular cylinders (radius= 10 mm, height= 5 or 10 mm, infill distance = 0.8 mm). Once printed, the paraffin molds were fitted into plastic flat-bottom microtubes, then infiltrated with hybrid sol obtained at Section 2.1, then centrifuged for 1 min to ensure penetration of the viscous hybrid sol into the void spaces of the paraffin template. Infiltrated molds were dried (for 3–5 days). Three consecutive overnight cyclohexane bathes were needed to dissolve the paraffin templates, and one overnight ethanol bath was needed to ensure complete removal of cyclohexane. The obtained porous BG/PCL hybrid scaffolds were finally dried for one day. Figure 1 shows an illustration of the synthesis and additive manufacturing process.

### 2.3. Bioactivity Evaluation in SBF Solution

The SBF (simulated body fluid) solution had ionic concentrations similar to human blood plasma and was produced as formulated by Bohner and Lemaitre [33]. Hybrid scaffolds previously prepared and dried were carefully cut into equal 1-mm-thick sections. Each section, averagely weighing 20–25 mg, was soaked in SBF (1 mg/mL). Samples were incubated at 37 °C on an orbital shaker (120 rpm) to avoid settling. After 1 h, 6 h, 1 day, 3, 7, and 14 days (21 days also in the first round of experiments), they were removed, then immersed in absolute ethanol to inhibit any further interaction/CaP formation. These interacted sections were kept for microscopy. Extracted SBF was filtered with 0.22 µm syringe filters at each time point.

### 2.4. ICP-OES (Inductively Coupled Plasma-Optical Emission Spectroscopy)

The concentrations of Ca, Zn, Si, and P ions released into the SBF medium were measured by ICP-OES. The mean values combined with standard deviations from triplicates for each dissolved ion were analyzed and recorded. Calibration solutions were prepared to obtain a linear correlation between ions’ intensity and concentration.

### 2.5. PIXE (Particle-Induced X-ray Emission) Analysis

Elemental composition of the non-soaked and SBF-soaked hybrid scaffold sections was also obtained by Particle-Induced X-ray Emission (PIXE) nuclear microprobe. This is an elemental non-destructive method, similar to Energy-Dispersive X-ray Spectroscopy (EDS) or X-ray Fluorescence (XRF), with increased sensitivity due to low Bremsstrahlung background and is capable of chemically mapping structures down to a submicronic resolution [34]. Samples were first embedded in epoxy resin (Agar 100 Resin, agar scientific, Stansted, UK), then cut into slices of about 150 µm thickness with a low-speed diamond saw. PIXE quantitative chemical imaging was then carried out at the AIFIRA platform (LP2I, UMR5797, Gradignan, France) using a 3 MeV incident proton beam (beam diameter of 1 µm).

An 80 mm^2^ lithium-doped silicon (Si(Li)) detector, commonly used in EDXS and low-energy gamma ray detection, equipped with a 12-μm-thick beryllium window and a 100-µm-thick aluminum “funny filter” (central hole diameter = 2 mm). Prior to the investigation of samples, soda-lime flat glass composed of ten oxides (NIST-620, USA) was used as a standard reference for calibration purposes. Conversion of X-ray peaks intensities to ion concentrations was carried out with Gupixwin software (Version 2.2.4, University of Guelph, Guelph, ON, Canada) to identify the local composition of desired ions.

### 2.6. Pair Distribution Function (PDF) Analysis

Local atomic structure of Si/Ca 75/25, Si/Zn 75/25, and Si/Ca/Zn 75/15/10 sol–gel-derived grounded BG powders have been measured with X-ray PDF analysis. All samples studied here were previously proved as amorphous according to their corresponding XRD patterns as perquisite prior to PDF analysis. The atomic PDF were obtained from X-ray total scattering data collected on a PANalytical Empyrean diffractometer equipped with a solid state GaliPIX3D detector, a focusing X-ray multilayer mirror, and an Ag anticathode (Kα1 = 0.5594214 Å, Kα2 = 0.5638120 Å). Powder samples were placed in glass capillaries of 0.7 mm diameter. An empty capillary of the same type was measured in the same way for background subtraction. Data were recorded over the 1–145° 2θ range, which corresponds to an accessible maximum value for the scattering vector Q max of 21.4 Å^−1^. Data merging, background subtraction, and Kα2 stripping were conducted using HighScore 5.1 Plus software provided by PANalytical Corporation (Malvern, UK). It was also used to generate a corrected and normalized total scattering structure functions S(Q) and consider the above-mentioned bulk chemical compositions. Finally, the PDF or G(r) was calculated from the Fourier transforms of S(Q) truncated at 20 Å^−1^. Since the silicon content is the same in all samples, the number of the Si-O pairs (direct interatomic distance) is expected to be the same in all samples; the PDF curves were thus scaled to obtain a similar intensity for the Si-O PDF peak at 1.62 Å. The simulated PDFs were calculated using PDFgui software version 1 [35].

### 2.7. Solid State ^29^Si Magic Angle Spinning Nuclear Magnetic Resonance (MAS-NMR) Spectroscopy

Q^n^ silicon species distribution inside the binary and ternary BG compositions (75/25 Si/Zn and 75/15/10 Si/Ca/Zn) were determined using a Bruker Avance I spectrometer operating at a magnetic field of 9.4 T (1H and ^29^Si Larmor frequencies of 400.2 and 79.5 MHz) using a 4 mm double resonance MAS probe head. The Si quantitative MAS spectra were recorded at a rotor spinning frequency of 10 kHz after a RF pulse of 2.4 µs (corresponding to a flip angle of 30°) and a recycle delay of 10 s. ^29^Si chemical shifts were referenced relative to tetramethylsilane. The spectra were simulated using DMfit software dm2011 (CEMHTI, CNRS, Orléans, France). The following equation was used to calculate the Degree of Condensation (DC) of BG:

(1) $$\mathrm{DC}\;=100\times \frac{2\times Q^{2}+3\times Q^{3}+4\times Q^{4}}{4}\;$$

### 2.8. Scanning Electron Microscopy (SEM)

Samples were carbon-coated prior to SEM observation and then the micro/macro porous structure, topography, and morphology of the scaffolds were observed with a field-emission gun scanning electron microscope Regulus 8230 (Hitachi, Japan) operating at 1kV. Images were recorded with a secondary electron detector. Alongside X-ray microanalysis was performed in areas of interest with an EDS Ultim Max 170 mm^2^ detector (Oxford, UK) at an electron accelerating voltage increased to 10 kV.

### 2.9. X-ray Diffraction (XRD)

Diffraction patterns were collected on a Philips X-Pert Pro equipped with a X’celerator 1D detector (2.122° active length) using CuKα1/Kα2 radiations (1.5406/1.5444 Å) in Bragg Brentano θ-θ geometry from 2 to 90° (2θ) with a scan step of 0.066°. Data analysis was performed with HighScore Plus software provided by PANalytical Corporation and Crystallography Open Database (COD) to identify the crystalline peaks. 

### 2.10. Fourier-Transform Infrared Spectroscopy (FTIR)

FTIR spectroscopy was used to assess scaffolds’ chemical functional groups before and after SBF immersion. The spectra were acquired with a Nicolet 380 FT-IR (Thermo Fisher Scientific, Schwerte, Germany) equipped with a diamond Attenuated Total Reflectance (ATR) accessory. Thirty-two spectral scans at a resolution of 4 cm^−1^ were repeated over the wavenumber range 4000–500 cm^−1^ (mid-IR region).

### 2.11. Cellular Assays

#### 2.11.1. Cell Culture

Cells were cultured with α-Minimal Essential Medium (PAN-Biotech GmbH, Aidenbach, Germany) supplemented with 10% fetal bovine serum (Anprotec, South Africa), 60 IU/mL penicillin, 60 µg/mL streptomycin, and 2.5 µg/mL amphotericin B as culture medium. Cells were maintained at 37 °C, high relative humidity, and 5% CO_2_. Cells from passages 3 to 5 were used in the experiments. All supplements were purchased from Sigma-Aldrich Co., St. Louis, MO, USA, if not mentioned otherwise. Falcons and Flasks were purchased from Sarstedt, Nümbrecht, Germany.

#### 2.11.2. Isolation of Bone Marrow Derived hMSCs

The hMSCs were isolated from the bone marrow of the femoral heads of 4 patients (3 male, 1 female; mean age 66,8 years; range 59–76 years) undergoing hip replacement surgery (Klinikum Großhadern, Munich, Germany). The study was approved by the LMU medical ethics committee (ID: 22-0379, 8-8-2022), with informed patient consent being required. After being transported in saline solution, bone marrow was scraped out and washed with 30 mL phosphate buffered saline (PBS, Biochrom, Berlin, Germany) before being poured through a cell strainer (pores size 70 µm, BD Bioscience, San Jose, CA, USA) into a 50 mL falcon. The remaining bone marrow pieces were digested three times for 10 min each on a 3D shaker in an incubator (37 °C) with 10 mL Collagenase II solution (1 g/1 mL in cell culture media). The 10 mL solution was poured into another falcon through a cell strainer (pore size 70 µm, BD Bioscience, San Jose, CA, USA). Both falcons were centrifuged (500 g, 5 min, RT), media was discarded, and each cell pellet was suspended in 10 mL cell culture media and transferred to culturing flasks. Following that, all flasks were kept in the incubator (Binder, Tuttlingen, Germany), and media was changed twice a week. Cells were passaged at 90% confluence. 

Multilineage differentiation was used to assess stem cell traits such as adipogenic, osteogenic, and chondrogenic differentiation potential, following the recently published protocol [36]. After several weeks, successful differentiation was demonstrated using Bodipy stainings, Alizarin Red staining, and Safranin O staining. Images were captured using a light microscope (Axioobserver, Zeiss, Oberkochen, Germany). Cells were also characterized with flow cytometry sample analysis for the expression of the CD73, CD90, and CD105 stem cell markers. After trypsinization and washing, 1 × 10⁶ cells were incubated in 100 μL staining volume with antibodies against CD73, CD90, and CD105, which are stem cell markers, and CD34 and CD45, which serve as a negative control [37,38]. For detailed information on staining reagents and antibody panel, a list is attached in Supplementary Information (see Table S1). After incubation on ice for 30 min and washing, flow cytometry measurements were performed with a BD LSRFortessa™ Cell Analyzer (BD Bioscience, San Jose, CA, USA). FlowJo™ v10.8.1 software (Becton, Dickinson and Company, Ashland, OR, USA) was used to analyze data.

#### 2.11.3. Indirect Cell Viability Assay with Cell Proliferation Reagent WST-1

Scaffolds (n = 2 per group) from each group [Hyb80 and Zn-Hyb80] (respectively, undoped and Zn-doped hybrid with PCL 80k) were incubated in cell culture media for up to 1, 5, or 7 days to determine the potential cytotoxicity of the scaffold material. Cell culture media was α-Minimal Essential Medium (PAN-Biotech GmbH, Aidenbach, Germany) supplemented with 10% fetal bovine serum (Anprotec, South Africa), 60 IU/mL penicillin, 60 µg/mL streptomycin, and 2.5 µg/mL amphotericin B (all Sigma-Aldrich Co., St. Louis, MO, USA). The extraction media (EM) was collected and stored at −80 °C. Cells were cultured in 96-well plates (Cellstar^®^, Greiner Bio-One GmbH, Frickenhausen, Germany), at a density of 10⁴ cells/well and cultured under addition of EM. As a negative control, cells were treated with normal cell culture media. After 24 h, cells were washed with PBS to remove EM and cell proliferation reagent WST-1 (Roche Diagnostics GmbH, Mannheim, Germany) was added (1:10 dilution) and incubated for 3.5 h. All quantifying experiments (indirect cell viability assay) were confirmed with four biological donors and with at least three independent experiments performed for each donor. A scanning microplate reader was used to measure absorbance at 450 nm (Multiskan FC, Thermofisher Scientific, Schwerte, Germany). The wavelength 620 nm was chosen as a reference. An illustration of the experimental procedure is schematically shown (Figure S1).

#### 2.11.4. Seeding and Cultivation of the Scaffolds 

The hMSCs were expanded up to passage 5 and then drop-seeded at a density of 2 × 10^5^ cells/scaffold. Five drops of 5 mL each were pipetted onto the scaffolds, after which the scaffolds were incubated for 5 h with an hourly moisture check. Then, 5–20 mL culture media was added as needed. Cell culture media was added after 5 h of incubation. The media was changed three times a week until the cells were confluent, then the media was changed to twice a week osteogenic media (α-MEM supplemented with 10% FBS, 60 IU/mL penicillin, 60 g/mL streptomycin, 100 nM dexamethasone, 10 mM glycerophosphate, and 0.17 mM ascorbate-2-phosphate). Three-dimensional constructs were cultured for 7 weeks in total.

#### 2.11.5. LIVE/DEAD Staining

The cytocompatibility of the scaffolds was further assessed by a LIVE/DEAD staining. Cells on the scaffolds were stained with 10 µg/mL fluorescein diacetate (FDA) in PBS and 1 mg/mL Propidiumiodide in PBS at 3, 5, and 7 weeks. Based on intracellular esterase activity, viable cells convert FDA to green fluorescein, which stains viable cells green. As a nucleic acid dye, propidiumiodide can only enter compromised cell membranes of dying or dead cells, dyeing them red. Pictures were taken with a Leica SP8 WLL confocal microscope (Leica Microsystems GmbH, Wetzlar, Germany). Non-quantifying experiments were confirmed with two biological donors.

#### 2.11.6. Statistical Evaluation

Descriptive analyses were performed with GraphPad Prism 9.4.0 (673) (GraphPad Software, La Jolla, CA, USA). Statistical inferences regarding the indirect cell viability assay were based on random intercept models using the GLIMMIX procedure of the Statistical Analysis System SAS release 9.04.01M6P11072018 for Linux (SAS Institute, Cary, NC, USA). Fixed effects were material itself, time of incubation with the material, and interaction between material and length of incubation. Post-hoc tests were adjusted for multiple testing by multiplying the uncorrected *p* value by the number of tests (Bonferroni correction). Adjusted *p* values < 0.05 were considered statistically significant. 

## 3. Results

### 3.1. NMR and PDF Analysis of Si/Zn and Si/Ca/Zn BGs

**Figure 2:**
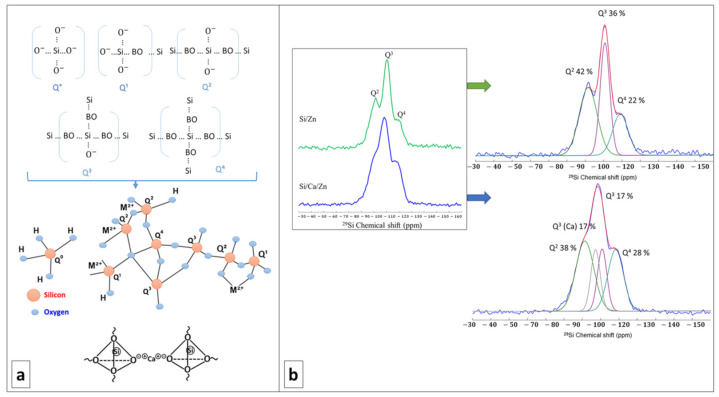
(**a**) Up: schematic representation of different Qn species in a silicate network (BO = Bridging Oxygen); down: disordering the network by modifier cations, e.g., Ca, M^2+^represents bivalent metal cations introduced to the bioactive glass network. (**b**) 29Si MAS-NMR spectra of Si/Zn (top) and Si/Ca/Zn (bottom) glasses and their deconvolution. Individual Qn contributions are shown in Table 1.

The Q^n^ species distribution was extracted from the ^29^Si MAS-NMR spectra visible in Figure 2. The respective proportion of each species, along with the degree of condensations of Si/Ca/Zn and Si/Zn, is brought in Table 1. Q^n^ refers to Si atoms bonded to n bridging oxygen atoms in a silicate network, with bridging oxygen (BO) being shared between two silica tetrahedra (Si-O-Si) (Figure 2a). Therefore, the more Q^4^ species, the more polymerized and condensed the network, as expected from a pure silicate network. Non-bridging oxygen (NBO) atoms can either arise from the silanol groups (Si-OH) or from network modifier cations (alkaline or alkaline earth cations as Ca^2^⁺ or Zn^2^⁺ in our case, as shown in Figure 2a) which both disrupt the connections between silica tetrahedra. The more Q^2^ and Q^3^ species, the more depolymerized the silicate network. 

In our case, the presence of Q^2^ and Q^3^ species is in favor of successful incorporation of network modifiers. This investigation of cation incorporation is of primary importance when soft chemistry routes are used and thermal treatments are banished like in the present work, as, e.g., the temperature at which Ca is incorporated into a silicate network was found to vary between RT and several hundred degrees depending on the calcium source [39]. From Figure 2b and the deconvolution of ^29^Si MAS-NMR spectra, it is visible that both Si/Zn and Si/Ca/Zn BG networks are poorly polymerized, with an abundant proportion of Q^2^ and Q^3^ species. For the ternary Si/Ca/Zn BG, it is possible to identify a characteristic signature of Ca incorporation with the presence of Q^3^(Ca) species. However, no specific signature of Zn incorporation can be identified in the spectra, although it could account for the observed proportion of Q^2^ and Q^3^ species (if Zn acts as a network modifier). Nearly identical DCs (Table 1) for the Si/Zn and Si/Ca/Zn BGs are calculated, indicating the same degree of disruption in silicate glass networks disrespectful of the doping percentage of the glass with zinc. The DC is also close to the one reported for the binary Si/Ca system in a previous work [40].

**Figure 3:**
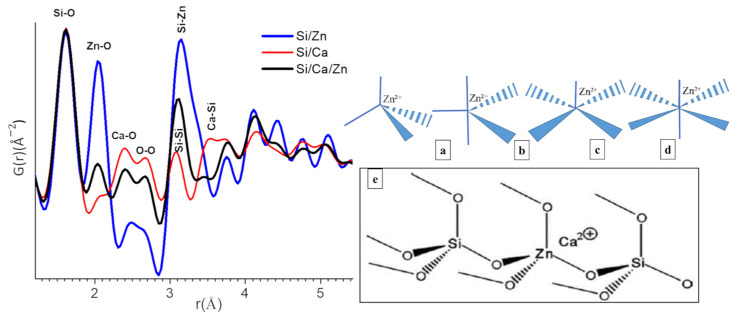
**Left**: the pair distribution function (PDF) curve obtained from total X-ray scattering data from different bioactive glass samples (respective compositions for blue: Si/Zn 75/25, red: Si/Ca 75/25 and black: Si/Ca/Zn 75/15/10 (all relative atom%)). **Right**: Zn-O coordination structures (**a**) tetrahedral (C4), (**b**) trigonal bipyramidal (C5), (**c**) square pyramidal (C5´), (**d**) octahedral (C6). (**e**) Structural model for Zn cations within the silicate network as tetrahedral ZnO_4_ unites and charge-balanced by Ca ions, as proposed by Shahrabi et al. [44].

Extraction of the data from XRD analysis depends mainly on Bragg peaks and we like them to be narrow, fully separated, and well representative; however, any sort of deficiencies or lack of any long-range order makes it a challenge. As shown by research groups, including Proffen et al. [41,42], the PDF analysis is an interesting method to investigate poorly crystalline and even amorphous systems. The pair distribution functions G(r) for r-values up to 5 Å are given in Figure 3 (left). The low crystallinity of the present samples yields atomic PDF that rapidly decays to zero at distances of 5–10 Å but still allows studying local atomic arrangement in materials. Indeed, the peaks on the PDF observed below 5 Å correspond to an interatomic distance between the first and second neighboring atoms while the intensity is proportional to the number of pairs at a given distance or the coordination number in the different shells and to the product of the scattering powers of the atoms forming the pair. The width of the peaks can give information about the distribution of the distances; the broader the peak, the larger the distribution of distances, which can come from disorders. As a function of the nominal chemical bioactive glass, there are changes in all atom-atom correlations shown in Figure 3 (left). Since all the contributions of all pairs of atoms are considered in the PDF, it can be useful to refer to structural models/reference samples to determine to which pairs these correlations belong. In the present case, we referred to SiO_2_ (quartz-type; COD 1526860) and ZnO (zincite-type; COD 1011258) structural models (Figure S4) and also data reported for calcium silicate hydrate (C-S-H) gels [43]. Based on this model, we were able to assign the peaks of PDF curve as follows: r = 1.62, 2.05, 2.39, 2.66, 3.08, 3.14, 3.54 Å to Si-O, Zn-O, Ca-O, O-O, Si-Si/Si-Zn, and Ca-Si contributions. All atom-atom correlations are worked out and mentioned in Figure 3 (left). Despite major differences in synthesis methods, it is interesting to note that the short-range order of C-S-H gels mostly resembles to our bioactive glasses; particularly, Si-O and Ca-O atom-atom distances are found nearly at the same positions [43]. Ca incorporation into Si/Ca and Si/Ca/Zn BG silicate network is evidenced by the presence of Ca-O correlations at 2.39 Å and Ca-Si distance observed at 3.54 Å for Si/Ca BG. Both distances obviously disappear in Si/Zn, which does not contain Ca. On the other hand, in ternary Si/Ca/Zn BG, the Ca-O distance is maintained and the Ca-Si distance disappears, suggesting that Ca is more distant/is farther from Si when Zn is introduced in the glass structure [43]. 

### 3.2. Primary Morphological Observations 

**Figure 4:**
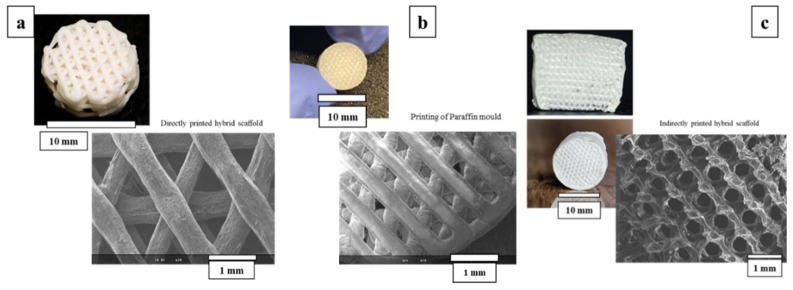
Imaging and SEM observations of the (**a**) direct printing method of the hybrids. (**b**) Paraffin template and (**c**) indirect printing of the hybrids.

A schematic presentation of printed paraffin template, obtained with indirectly printed scaffolds and directly printed scaffolds besides geometrical and morphological features as observed in SEM, is shown in Figure 4. The mean strut diameter for the direct printed scaffolds is 0.547 ± 0.038 mm and the maximum width equal to about 0.607 mm is usually observed at the contact point of the strand with its beneath strand. Widening is observed in all these node-like places whereas in-between the next node, we can see the thinning of the strand. Inter-strand distance is 0.650 mm. Considering the indirect printing method, the images of negative paraffin mold indicate the mean strut value of 0.371 ± 0.021 mm and an inter-strut space of 0.290 ± 0.006 mm.

### 3.3. Directly Printed vs. Indirectly Printed Scaffolds Reactivity of Zn-BG/PCL of 45k Hybrids

**Figure 5:**
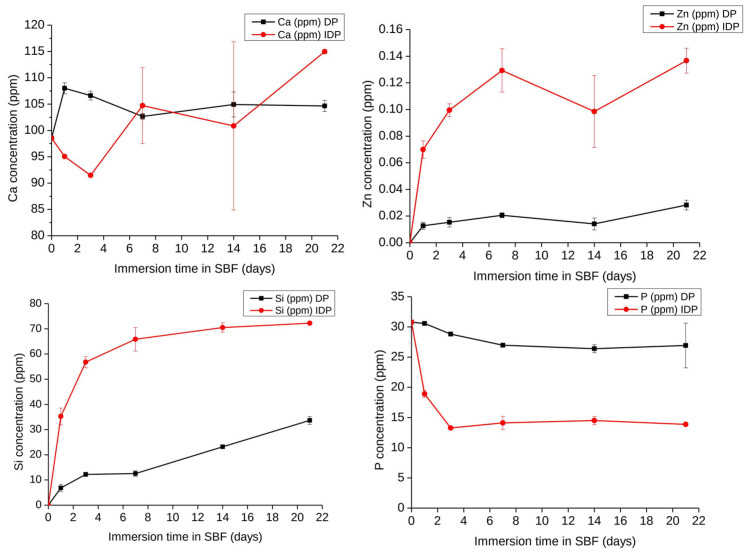
Evolution of Si, Ca, Zn, and P concentrations in Simulated Body Fluid during the immersion of Zn-BG/PCL 45k hybrid scaffolds (DP: Directly printed, IDP: Indirectly printed) (1 mg/1 mL SBF, 37 °C) as determined by ICP-OES, n = 3.

Over a three-week time interval, the ionic release behavior of directly and indirectly printed scaffolds was investigated as reported in Figure 5. Looking at the Si release in SBF, indirectly printed scaffolds degrade quicker in comparison with directly printed scaffolds. A similar behavior is found for Zn release, with surprising low Zn concentrations being released in SBF: a maximum of 0.14 ppm is reached for indirectly printed scaffolds, while no more than 0.02 ppm Zn are detected in the fluids for directly printed scaffolds. Regarding Ca, the trends remain unclear since Ca can be both released and its concentration increased in the medium as a result of the material degradation, or decreased as a result of CaP precipitation at the surface of hybrids. Therefore, the evolution of P in SBF is a good indication of the apatite-forming ability of the material. For the directly printed scaffolds, a slight decrease corresponded to a few ppm loss in P, while for the indirect printed scaffolds, the concentration of P was divided by two after three days of interaction, followed by a constant plateau up to the whole 21 days, suggesting no significant evolution of CaP formation or apatite formation being hindered in the long term. Obviously, the thinner strands of the indirectly printed scaffold increase the surface reaction and speed up the ionic leaching and exchanges and degradation of the material.

**Figure 6:**
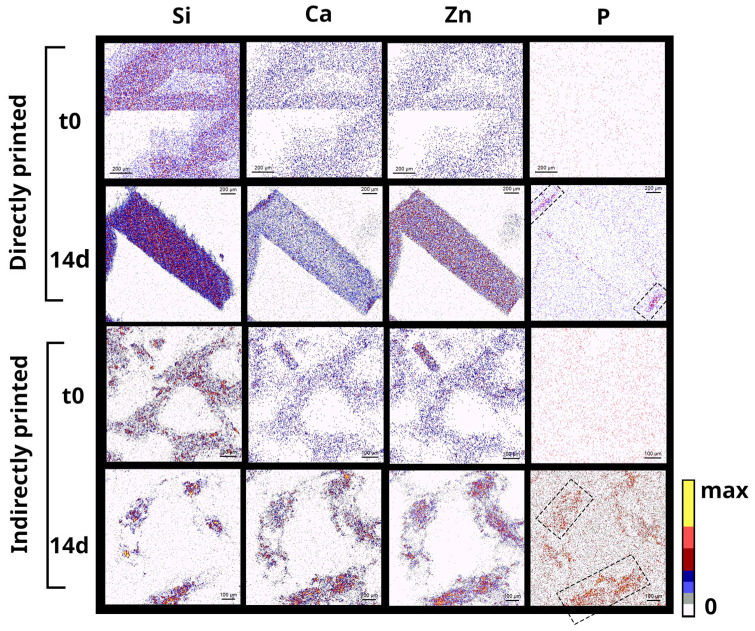
Cross-sectional PIXE chemical mapping of Si, Ca, Zn, and P in Zn-BG/PCL 45k hybrid scaffolds printed differently via direct and indirect routes each before and after immersion in SBF for 14 days (scale bars are 200 µm in two first lines and 100 µm in below ones).

Chemical distributions of elements inside the scaffolds in the starting materials and after 14 days of interaction with SBF are depicted in Figure 6. The ability to form CaP is evidenced for both type of scaffolds. For directly printed scaffolds, CaP remain deposited within a thin layer at the surface of the strands, while for indirectly printed, P seems quite evenly distributed within the strands, showing an extended CaP deposition through the whole material. Zn and P maps overlap in mineralized areas, indicating the colocalization of CaP depositions with Zn in the samples.

Considered simultaneously with the ICP-OES measurements, the chemical distribution in the scaffolds show that the indirectly printed scaffolds are superior in terms of bioactivity and Hap formation. However, the indirectly printed scaffolds were extremely fragile and subjected to premature degradation in SBF solution. Observing severe brittleness for the indirectly printed scaffolds and poor bioactivity potential of directly printed scaffolds led us to opt for the indirectly printing technique but using a higher molecular weight (average Mn 80,000; PCL80k instead of lately 45k). Afterwards, these will be mentioned as Zn-Hyb80 and Hyb80, respectively, in the following sections.

### 3.4. ATR and XRD Results on Zn-Hyb80 and Hyb80 Scaffolds

**Figure 7:**
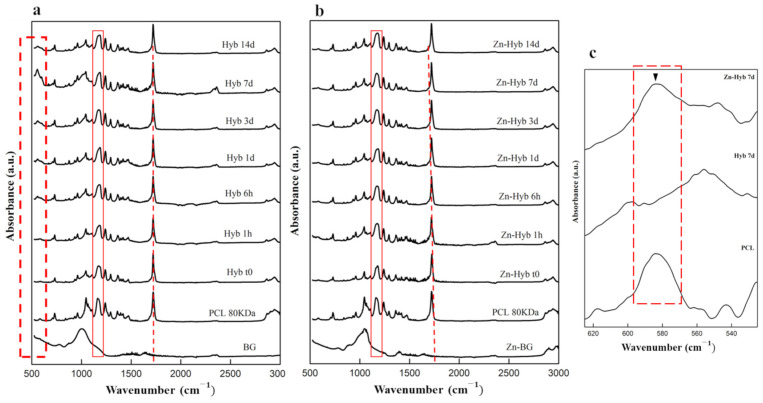
Evaluation of the apatite-forming ability of BG-PCL hybrid scaffolds. ATR spectra of (**a**) Hyb80 scaffolds and (**b**) Zn-Hyb80 scaffolds before and after immersion in SBF for 6 h, 1, 3, 7, and 14 days (1 mL of SBF per mg, 37 °C). (**c**) Comparison between the FTIR spectra of Hyb80 and Zn-Hyb80 after 7 days of immersion in SBF indicating nanocrystalline non-stoichiometric apatite formation highlighted by ▼ in dashed zoomed zone.

Pre-immersion spectra of both Zn-Hyb80 and Hyb80 scaffolds exhibit features of symmetric and asymmetric CH2 bands corresponding to peaks positioned at around 2863 cm^−1^ and 2942 cm^−1^ (3000–2800) cm^−1^ (Figure 7a,b), respectively [45]. The most intense band at 1720 cm^−1^ is correlated to carbonyl stretching ν(C=O). This carbon-oxygen esteric stretching band is observed in all the other graphs and they are normalized based on this peak. The next band at 1293 cm^−1^ is indicator of C-O and C-C stretching. The band observed at 1240 cm^−1^ correlates to asymmetric C-O-C stretching and the one at 1175 cm^−1^ corresponds to ester C-O stretching. The band shown in a rectangular red zone highlighted in both diagrams of Zn-Hyb80 and Hyb80 can be ascribed to C-O stretching. All these peaks are in perfect agreement with the characteristic peaks of the polymer PCL reported elsewhere [46].

The first ATR pattern positioned at the bottom of each stack corresponds to the raw BG. In case of Hyb80, the only band peak positioned at 1004 cm^−1^ is ascribed to Si–O–Si asymmetric stretching. In search of signs confirming the apatite formation, the characteristic band peak positioned at 962 ± 2 cm^−1^, despite its insignificant intensity in IR spectroscopy, is an interesting proof (Figure 7c). This band representing the ν_1_(PO_4_)^3−^ is reported at same position for apatite phases or OCP, but in the case of amorphous calcium phosphate, monetite, or brushite is shifted to 950, 995, and 985 cm^−1^, respectively [47].

**Figure 8:**
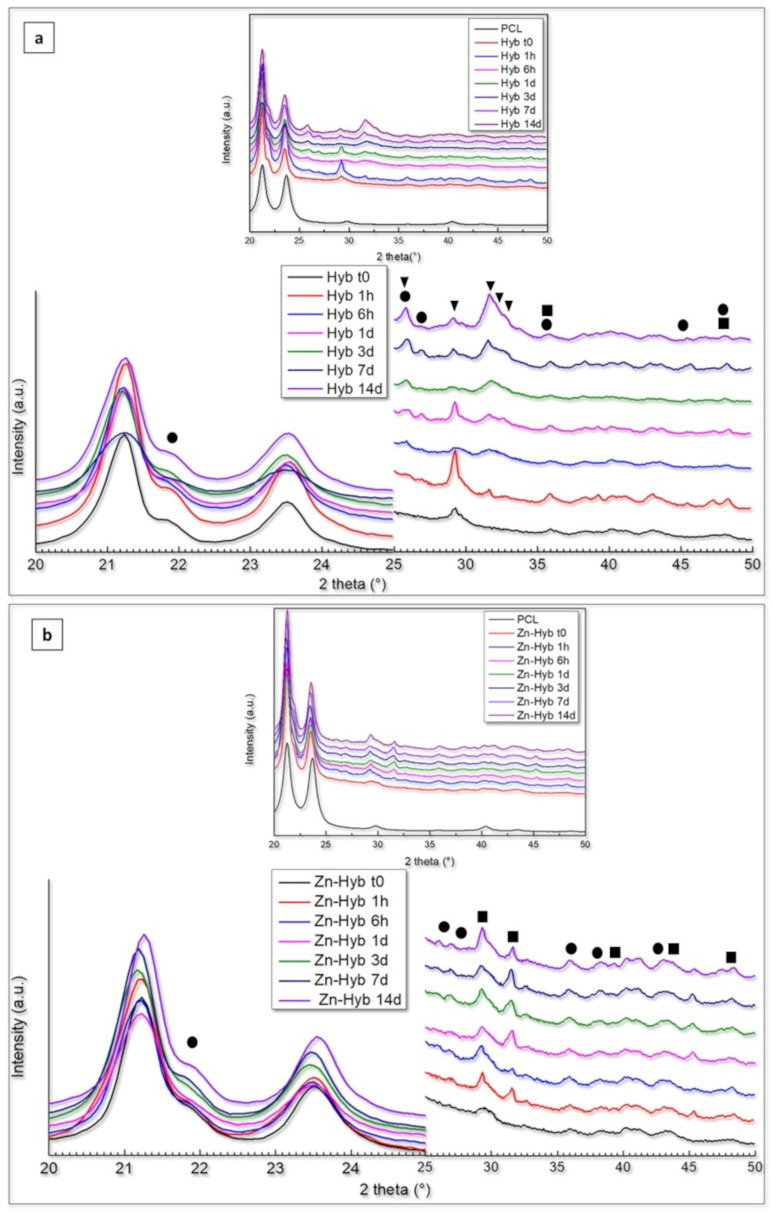
(**a**) XRD patterns of Hyb80 scaffolds, (**b**) XRD patterns of Zn-Hyb80 scaffolds after determined time intervals of immersion in SBF (▼: HCA/Hap feature peaks, ■: Calcite, ●: Aragonite).

The XRD results of 3D indirectly printed scaffolds of Hyb80 and Zn-Hyb80 are presented in Figure 8. Primarily, there are two very sharp peaks at about 21.24° and 23.68° (2θ) attributed to PCL, respectively corresponding to the (110) and (200) planes of the orthorhombic crystal structure [48]. An additional peak positioned at 29.7° (2θ) is observed, which is related to alignment of the polymer chains because of tetrahydrofuran evaporation and printing process (preferred orientation). Due to the amorphous nature of the bioactive glass component, all the other non-PCL characteristic peaks are related to newly formed phases after immersion in SBF. The patterns of the phases formed after the immersion disclosed broad peaks that can be related to their low crystallinity and/or small grain sizes [49]. A shoulder feature positioned at 22.06° is observed in all Zn-Hyb80 and Hyb80 samples, revealing the formation of aragonite phase and corresponding to the (002) plane of its orthorhombic lattice. 

In Hyb80 scaffold patterns, the formation of HCA (generic formula Ca_10–x_(PO_4_)_6–x_(CO_3_, HPO_4_)_x_(OH)_2–x_), calcite, and aragonite (CaCO_3_) phases was evident during SBF immersion. The observation of characteristic double peak at 25.96°/26.88° (2θ) along with the peaks positioned at 30.56°, 35.62°, 36.09°, 37.99°, 40.99°, 42.31°, and 45.47° (aragonite JCPD, reference code: 96-901-5562) support the presumption of an aragonite phase. On the other hand, Hap/HCA characteristic triplex peaks are detected at 31.69°/32.16°/32.81° (2θ), respectively corresponding to d-spacing planes of (121), (112), and (030).

### 3.5. SEM/EDS of Zn-Hyb80 and Hyb80 Scaffolds 

**Figure 9:**
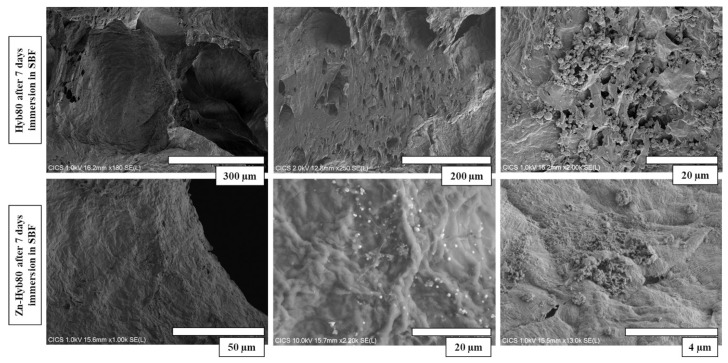
Microstructure of Hyb80 and Zn-Hyb80 scaffolds after 7 days of incubation in SBF. Images obtained by secondary electrons at accelerating voltage of 10 kV. (More dispersion of post-immersion precipitation deposits on Hyb80 compared to Zn-Hyb80).

Polygonal crystals are fully covered on non-doped Hyb-80 scaffold after 7 days of immersion in SBF. Less precipitates were observed at the surface of Zn-Hyb80. It is noticeable that the sample doped with Zn exhibited less porosity on the surface and internal pore walls (Figure 9). SEM observation in Figure 9 and EDS elemental analysis (Figure S3) show the presence of calcite polymorphs, coexisting with CaP precipitates, as calcium and phosphorous are detected in most of the nucleation sites. Such proofs are not observed for Zn-Hyb80, whose bioactivity seems strongly diminished.

### 3.6. Ion Release in Mueller Hinton (MH) Media Compared to SBF

**Figure 10:**
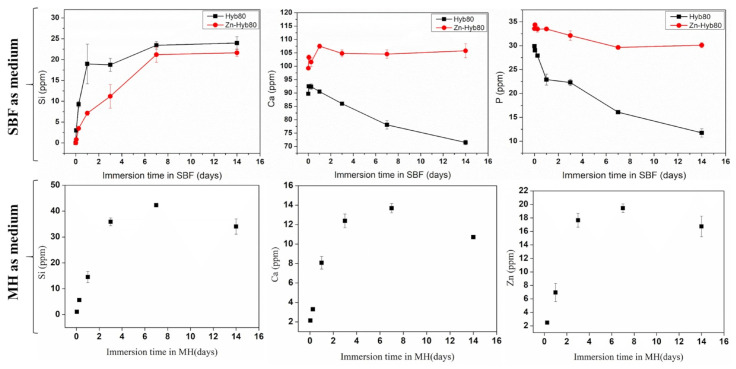
**Up**: ICP-OES measured evolution of the Si, Ca, and P of the Hyb80 and Zn-Hyb80 immersed in SBF up to 14 days (Zn always below 0.5 ppm is not included). **Down**: Evolution of Si, Ca, and Zn concentrations in Mueller Hinton Medium during the immersion of Zn-Hyb80 scaffolds (1 mL of MH per mg, 37 °C) as determined by ICP-OES, n = 3.

Given the very low release of Zn ion in SBF, we wanted to investigate the Zn-Hyb80 behavior in a protein-containing solution. The interaction between metals and proteins, including the formation of metallocomplexes, is of interest for its potential to increase the Zn bioavailability. The Mueller Hinton (MH) media is a yellowish transparent liquid commonly used in microbiological assays. The media is composed of beef extract, casein hydrolysate, starch, agar, and distilled water. The Zn-Hyb80 scaffolds were immersed in MH for 1 h, 6 h, 1, 3, 7, and 14 days and the ionic release was determined by ICP-OES. For comparison, the same was conducted in SBF for Hyb80 and Zn-Hyb80. In Figure 10, the decrease in P concentration in SBF confirms the materials are forming apatite but lowers when the hybrid is doped with Zn. As can be seen in Figure 10, in MH we see a high Si, Ca release and a remarkable increase in Zn release, with Zn concentration about 130 times higher than that measured in SBF (always below 0.5 ppm). This could be attributed to proteins’ ability to enhance the dissolution of ZnO.

### 3.7. Isolated Cells Prove Stemness and Multilineage Differentiation

All donors included in the study underwent multilineage differentiation successfully comparable to published data [36]. Flow cytometry characterization shows high expression for all stem cell markers (<99%) for the stem cells, with only one exception for Donor D, where CD105 expression was 62.5% (see Table S2 with dot plots for visualization). It has been reported that in bone-marrow-derived stem cells, CD105 negative subpopulations can be found, which is associated with even higher osteogenic differentiation capacity [50]. Minor subsets of cells are also CD34- and CD45-positive, which are markers for hematopoietic precursor cells. We assumed that direct isolation of bone marrow derived stem cells yielded different smaller subpopulations from the bone marrow including hematopoietic precursor cells.

### 3.8. Hybrid Materials Show Promising Cytocompatibility with hMSCs under Normal Experimental Conditions

**Figure 11:**
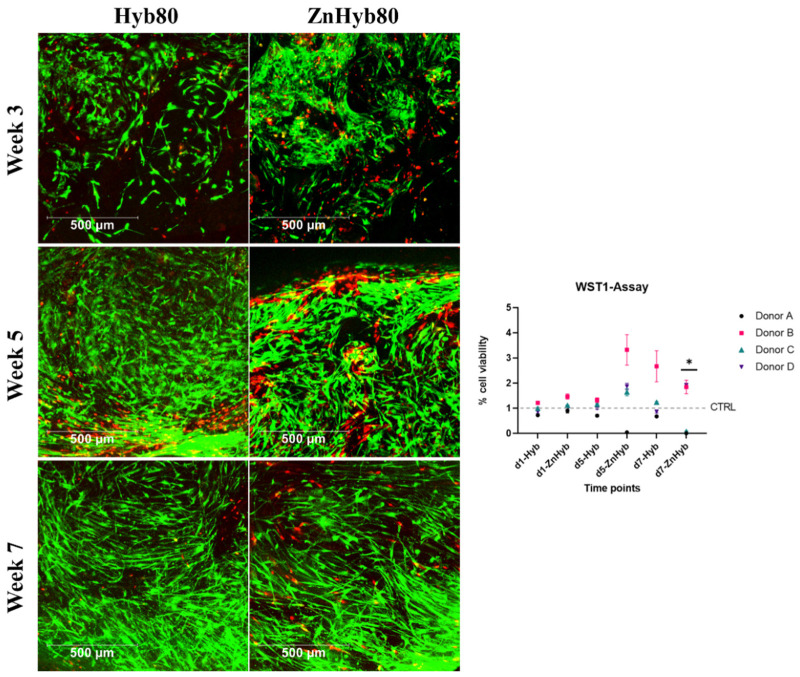
**Left**, LIVE/DEAD Staining of 3D constructs at 3, 5, and 7 weeks. Scale bars are 500 µm. **Right**: WST-1 indirect cell viability assay, all data are normalized against the control group (treated with normal cell culture media) and expressed as arithmetic mean ± standard error; * statistically significant difference between d7-ZnHyb and all other groups (*p* < 0.001 since *p* values were adjusted for multiple testing).

The indirect cell viability assay is used to assess the cytocompatibility of the hybrid materials. WST-1 is a tetrazolium salt that is converted to formazan dye by cellular enzymes. The amount of formazan product correlates with the number of metabolically active cells, making it a useful reagent for spectrophotometric cell number quantification. WST-1 is cleaved by metabolically active cells into formazan dye, which is measured spectrophotometrically with an ELISA reader. Any increase or decrease in measured absorbance directly correlates with the number of metabolically active cells, provided the seeding density is between 0.1 and 5 × 104 cells/well. Incubating EM with scaffolds for 1 day, 5 days, or 7 days results in an increasing proportion of released scaffold products. The indirect cell viability assay (see Figure 11) reveals no significant difference in absorbance between Hyb80 and control groups for 1, 5, and 7 days for all biological donors. This suggests excellent cytocompatibility for the undoped hybrid group for all time points since there is no significant change in the number of metabolically active cells. Similarly, EM incubated for 1 d with Zn-Hyb80 scaffolds leads to no decrease in the number of metabolically active cells, which implies that Zn-Hyb80 EM 1d had no cytotoxic effects.

The data for EM from the Zn-Hyb80 group incubated for 5 and 7 days with scaffolds were donor-dependent. For donor B and D, there is no significant difference in absorbance compared to the control group, i.e., not showing a cytotoxic effect for EM. The situation is different with donor A and C, where there is little to no absorption at EM of the Zn-Hyb80 group of 5 d and 7 d (Donor A) or 7 d (Donor C). This suggests a lack of metabolically active cells and thus either a decrease in cell number or an inhibitory influence on cell metabolism. The EM of the Zn-Hyb80 material with the higher concentrations of soluble products seems to have a donor-dependent effect on cell viability.

### 3.9. Scaffolds Allow Mid-Term Cell Culture for up to 7 Weeks with Excellent Cell Viability and Proliferation

In the LIVE/DEAD staining, viable cells can be detected on both scaffolds at all of the time points for both donors (see Figure 11 and Multimedia File S1 in Supplementary Information, showing a 3D image of cell ingrowth into the scaffold’s pores), indicating that both scaffold materials allow mid-term cell culture for at least 7 weeks with excellent cell viability and proliferation, indicating no change in cytocompatibility compared to control scaffolds. Over time, there is a visible increase in cell count and cell ingrowth into pores. A greater number of red marked dead cells is visible in the area of strong cellular ingrowth on the ZnHyb80 scaffold after 5 weeks of culture compared to the Hyb80 scaffold LIVE/DEAD staining can, however, not be considered suitable for quantification in this experimental setting, since only small parts of the scaffolds can be imaged and drop-seeding of scaffolds does not guarantee a homogenous cell layer.

## 4. Discussion

This study aimed at evaluating the impact of incorporating Zn inside hybrids consisting of BG and PCL. The materials were synthesized using an acid-base catalyzed sol–gel route, the BG phase being doped with Zn. Hybrids were additively manufactured through the direct printing of hybrid granules or indirect printing of the sol involving a 3D sacrificial template. Insights into the atomic structure of the BG were gained through solid-state NMR and X-ray PDF analyses, the former evidencing a limited impact of Zn incorporation on the silicate network itself and calcium incorporation, while the latter suggests an ambivalent role of Zn due to the tetrahedral coordination of ZnO that could act as a bridge between silicate tetrahedra. Combined, the NMR and PDF analyses suggest doping of the SiO_2_/CaO bioactive glass system with Zn leads to a structural change, which is not a mere substitution of Ca by Zn. From their ‘role in BG network’ point of view, Ca is known as a network modifying cation in the glass structure that produces non-bridging oxygen atoms, while ZnO is known to be capable of serving both as a network modifier and as an intermediate oxide depending on its proportion, with some supports for the former being gained by the ^29^Si MAS-NMR analysis, and supports for the latter being gained from the PDF analysis as discussed in Section 3.1. Therefore, at least a fraction of Zn is present in the form of tetrahedral species (ZnO_4_^2−^) that play an ambivalent role, capable of connecting silica tetrahedra [51], but these species need Ca ions to stabilize and balance the charge [44]. This could explain the reduction observed in the peak corresponding to “short” Si-Ca distances in the PDF of Si/Ca/Zn BG, along with the decrease in the rate of dissolution of the silicate network in SBF as observed with ICP-OES measurements. Accordingly, bioactivity is simultaneously decreased and delayed with Zn substitution for Ca, because of both the impact of Zn on the silicate structure and the lower amount of Ca in the glass for Si/Ca/Zn BG. 

XRD and ATR studies collectively point out a tedious decreased ion exchange with the immersion media for Zn-Hyb80. The same observations of reduction in the leaching activity of Zn-doped glasses are reported elsewhere [44,52].

In vitro apatite-forming ability tests in SBF confirm the ion release along with the hybrid’s bone-like apatite-forming ability. The presence of Zn ions in the bioactive glass structure is supposed to potentially lead into Zn-doped calcium phosphates. Unlike Hyb-80, traces of HCA formation are scarcely found in Zn-Hyb80 scaffold, even after longer periods of SBF immersion. Our results are consistent with previous studies about zinc inhibiting role on crystal formation/growth of CaPs. In fact, Legeros et al. found that Zn negatively affects de novo apatite formation and even remarkably diminishes the crystal size. At concentrations higher than 2 mM/L (130.76 ppm), Zn in solution even promotes the formation of more soluble CaP phases and mostly ACP instead of all the other multiple possible phases [53]. Other studies report a strong inhibition of Zn ions against apatite formation in vitro even up to 31 days of SBF immersion. The formation of CaPs was evidenced in Section 3.3 and Figure 6, but here the XRD analysis suggest that it remains in an amorphous state for Zn-Hyb80. The lower bioactivity we can conclude for the Zn-Hyb80 can be ascribed to the lower bioactivity of Zn-BG compared to binary BG. Zn is reported recurrently as an ion inhibiting the ion exchange with the surrounding media since it rivals the Ca ions and exceeds them in the formation of a Zinc calcium phosphate precipitation, thus mostly retarding any HCA precipitation. Moreover, the lower bioactivity could also be related to the Zn insertion in the silicate network. The infra-red peak positioned at about 960 cm^−1^ as a Raman shift in Raman spectroscopy clearly reveals the phosphate vibrational bands with major intensities [54]. This could be suggested for further investigations. However, here in IR spectra of our hybrid, the overlap of PCL and post-immersion hybrids in this same position makes it obscure to surely relate this band to CaP functional groups. Magnification of the ν_4_(PO_4_)^3-^ band positioned at 550 and 600 cm^−1^ is shown in Figure 7c, as well as its evolution during the immersion time in SBF solution. These bands can be an indicator of biological Hap formation and are observed in soaked Hyb80 IR after 7 days of immersion in SBF while they are not observable after 7 days of immersion for Zn-Hyb80. A feature of HCA formation is the asymmetric stretching vibration positioned at a wavenumber of about 1030 cm^−1^; however, in both Zn-Hyb80 and Hyb80 scaffold samples IR spectra, the PCL peak at about 1045 cm^−1^ is overlapping at all time points, hindering a deduction in having P-O asymmetric stretching proof. P-O bending vibration at estimate an wavenumber of 600 cm^−1^ in post-immersion patterns of Hyb80 is also a sign of Hap formation and, hence, bioactivity (Figure 7c).

Regarding Zn insertion in the silicate network, a classification of different possible Zn sites in the primary coordination sphere is shown in Figure 3 (right), forming 4, 5, or 6 coordination complexes. The Zn-O distance observed in 2.05 Å here is typical of a tetrahedral environment (four coordination complex), which is in agreement with the most prevalent option mentioned in the literature, namely tetrahedral [55]. Interestingly, the gradual shift in the peak maximum attributed to Si-Si/Si-Zn correlations to longer distances when increasing zinc content (3.08 Å for Si/Ca 75/25, 3.10 Å Si/Ca/Zn 75/15/10, and 3.14 Å for Si/Zn 75/25); further, simultaneous increase in the intensity of this peak and its broadening strongly suggest that at least part of these [ZnO_4_] tetrahedra are connected to the silicate network, as shown in Figure 3e. It may be recalled that in the case of zincite, the distance between neighboring is 3.20 Å (COD 1011258).

The release kinetics of Zinc differ significantly between directly and indirect 3D printed scaffolds. The structure obtained with 3D direct printing is denser because it results from coarse (several hundred µm) printing resolution and hot extrusion associated with FDM, and requires longer soaking periods in SBF to degrade, while the 3D indirect template method leads to finer pore sizes and thinner struts with internal porosity generated as a result of solvent evaporation, therefore enhancing apatite nucleation. 

Remarkably, diffusion and accumulation of zinc are observed at the surface of both kinds of hybrids within the newly formed active CaP layer. Zn release was found to be dependent on the scaffold printing method but also on the medium, with Zn^2+^ concentrations released being two orders of magnitude higher in Muller–Hinton Broth bacterial culture medium compared to SBF, reinforcing observations from previous studies about the ability of proteins to enhance the dissolution of ZnO by creating highly soluble complexes. Regarding proteins’ ability to enhance the dissolution of ZnO, casein phosphopeptides are well known to improve zinc absorption and casein hydrolysate is responsible for maintaining the Zn in solution during Ca and P precipitation [56]. Zn ions interact with certain amino acids and phosphate groups on the protein’s surface as determined by spectroscopic techniques and electron microscopy imaging [57]. For example, Zn^2+^ might bind to the phosphate groups of serine, the histidine imidazole ring (mainly nitrogen), or to the deprotonated carboxyl groups of glutamic and aspartic acids. Zn^2+^ may participate in the cross-linking of two or more neighboring charged amino acids groups [57]. Zinc is involved in many biological processes and has unique coordination chemistry, allowing it to form stable aqua complexes in acidic aqueous solutions that can exchange water molecules when binding to other ligands. Its affinity for functional groups such as carboxyl and amino groups of proteins and subsequent formation of metal ligands is probably the reason why the Zn release is observed to be more than 100-fold in a protein medium such as MH, unlike a protein-free one such as SBF. This is a good finding promising that a significant amount of Zn^2+^ ions can be delivered in vivo [58].

As we aimed for secondary biological assets with fractional substitution of Ca source with Zn source, it was concluded that in the first place, the Zn-doped hybrid biomaterial and following scaffolds are of no toxicity to mesenchymal human cells under normal experimental conditions and can perform as well as simple binary Si/Ca bioactive glass derived hybrids. The maintenance of cytocompatibility for the hybrid material was expected since both undoped and doped bioactive glass, PCL, and their hybrid materials are reported to possess good biocompatibility in literature [32,59,60,61,62] and Zn-doped and undoped bioactive glass had shown a beneficial effect on bone formation [62,63]. Similar results are obtained for the LIVE/DEAD staining—on all scaffolds, the vast majority of cells are alive for up to 7 weeks. Over time, there is a visible increase in cell count and cell ingrowth into pores, indicating that the scaffolds possess excellent potential as tissue templates for 3D constructs and bone substitutes. Although there were some areas with increased red staining, especially if strong cellular ingrowth had been obtained, there was no trend observed towards a less pronounced cytocompatibility within doped hybrid scaffolds in general. Undoped and doped hybrid scaffolds showed the ability to function as templates for cells to attach, grow, and proliferate desirably, leading to bone remodeling.

In order to quantify any potential cytotoxic effects, WST-1 cell proliferation assays were conducted with EM. Undoped hybrid showed no cytotoxic effect for all time points for all biological donors since there were no statistically significant differences in the absorbance measurements compared to the other groups. This underlines the impressions from the LIVE/DEAD staining. Similarly, there were no statistically significant differences for Zn-Hyb80 EM for 1 d and no cytotoxic effects for the EM from 1d of doped hybrid scaffolds.

Regarding the EM from 5d and 7d, there are donor-dependent differences in the number of metabolically active cells. In vivo, Zn is considered a relatively non-toxic metal element that is only toxic to humans and animals at really high doses [64]. Still, free Zn ions outside a narrow concentration range have been shown to be cytotoxic to various cell lines and primary cells [65], demonstrating the oligodynamic effect of the metal Zn [66]. It may have good biocompatibility and antibacterial properties at low concentrations, but it also has cytotoxic effects at high concentrations [65]. Researchers demonstrated that biodegradability and extensive mass loss of the polymer matrix of composite-based scaffolds consisting of a bioceramic phase and a polymer phase at some time points could lead to high concentrations of release products, which may cause cell death [59,67]. Regarding the donor-specific data, we assume that there is inter-donor variability with Zn tolerance. Random intercept models were calculated to exclude donor-dependent Zn tolerance and only evaluate material-specific influences on cell viability. Only for 7d, the Zn-Hyb80 EM with the highest concentration of released products led to a significant decrease in the number of metabolically active cells in the random intercept model. This implied that only Zn-Hyb80 EM with relatively high concentration exhibited cytotoxic effects.

Limitations of this study are that in 3D printing the accuracy of the product is difficult to achieve at a nanometer scale [68] compared to electrospinning and electrowriting. In our case, we were looking for a method which offers a feasible approach to deliver a construct that offers low complexibility, but concentrating on the material question including options to transform into a ready-for-clinical-use status as soon as possible. Despite the advantages of the electrospinning method such as not activating the immune response, the 3D printed scaffolds offer good possibilities to evaluate composite materials on a first basis.

As cell culture on Zn-Hyb80 scaffolds included three media changes per week, incubating EM for 7 days with Zn-Hyb80 material would appear to be an experimental exaggeration of the real conditions. Nonetheless, potential cytotoxic effects at higher concentrations within the scaffold’s material cannot be excluded. Cytocompatibility is the most essential trait for novel biomaterials in order to facilitate bone regeneration and minimize tissue damage [5]. Overall, our findings show that Hyb80 and Zn-Hyb80 materials have excellent biocompatibility with good cell viability and proliferation on scaffolds for at least 7 weeks and have no adverse effect on cell viability under normal experimental conditions, making them good candidates for BTE.

## 5. Conclusions

This study was conducted to design novel Zn-doped hybrid materials consisting of BG and PCL. The performance of these scaffolds was evaluated through physicochemical characterization and in vitro 3D cell culture, showing promising release kinetics and excellent biocompatibility. Direct and indirect cellular assays involving hMSCs were conducted. Both groups of hybrid scaffolds offer the ability to perform as a mid-term host for cells to attach, grow, and proliferate desirably, leading to bone remodeling. The indirect cell viability assay displays no cytotoxic effects of the Zn-hybrid under normal experimental conditions. This is a major concern to be addressed given the relative sensitivity of eukaryote cells exposed to Zn and possible Zn^2+^ homeostasis deregulation. LIVE/DEAD staining of hMSCs 3D cultured in the scaffolds for prolonged periods demonstrate excellent biocompatibility for at least 7 weeks.

Overall, the hybrid material, either non-doped or doped with metal trace element, is a promising candidate to be translated to clinical applications for bone regeneration. Considering the antibacterial potential, the improved Zn ion release in protein-containing media can open new avenues in biomedical applications attached to different conditions.

## Figures and Tables

**Table 1:** Relative intensities of Q^n^ resonances obtained from fits and degrees of condensations (DC).

Si/Zn	Q Species	δ_iso_ (ppm)	FWHM (ppm)	%	Si/Ca/Z	Q Species	δ_iso_ (ppm)	FWHM (ppm)	%
	**Q^4^**	−109.1	10.0	22		**Q^4^**	−108.0	10.0	28
	**Q^3^**	−100.7	6.1	36		**Q^3^**	−100.6	6.0	17
	**Q^2^**	−91.5	10.0	42		**Q^3^(Ca)**	−97.0	6.0	17
						**Q^2^**	−91.2	11.6	38

DC (Si/Zn) = 70, DC (Si/Ca/Zn) = 72.

## Data Availability

Upon request, all data can be obtained from the corresponding authors.

## Supplementary Materials

The following supporting information can be downloaded at: https://www.mdpi.com/article/10.3390/cells12131759/s1, Figure S1: Indirect cell viability assay; Table S1: Staining reagents—antibody list; Multimedia file M1: LIVE/DEAD Staining of Hyb scaffold at 3 weeks; Figure S2: Flow cytometric analysis of stem cell marker expression—Donor C; Table S2: Flow cytometry analysis of bone marrow primary cells; Figure S3: SEM images and EDS spectra of (i) Hyb80 and (ii) Zn-Hyb80 scaffolds after 7 days of incubation in SBF; Figure S4: PDF simulations of Quartz (left: COD 1526860) and Zincite (right: COD 1011258); MultimediafileM1 Video S1: LIVE/DEAD Staining of Hyb scaffold at 3 weeks, Scale bar is 200 µm.
