# Supplementary material for: Zinc-Doped Bioactive Glass/Polycaprolactone Hybrid Scaffolds Manufactured by Direct and Indirect 3D Printing Methods for Bone Regeneration
Source: Cells. 2023 Jun 30;12(13):1759. doi: 10.3390/cells12131759 (PMC10341101; doi:10.3390/cells12131759)
Supplement: Supplementary file 1 [file cells-12-01759-s001.zip › cells-2432152-supplementary.pdf]

Supplementary Information

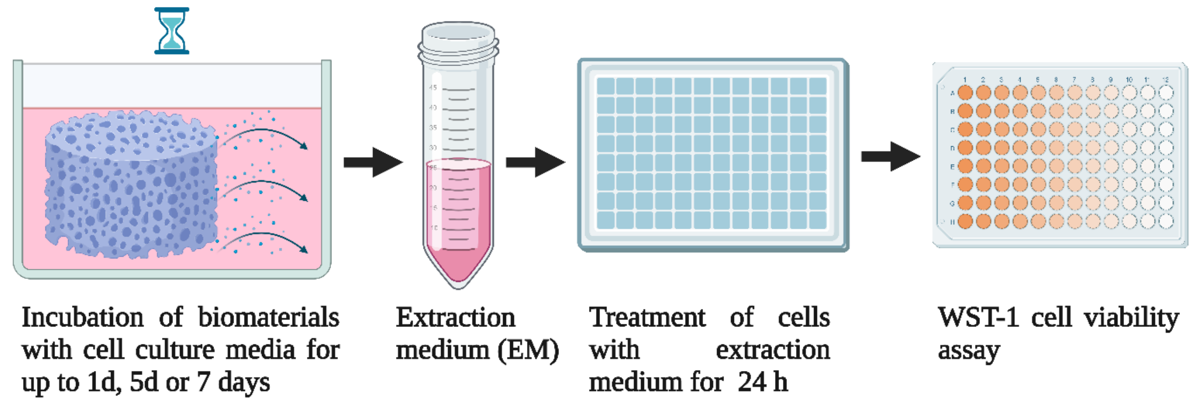

Figure S1. Indirect cell viability assay, created with BioRender.com.

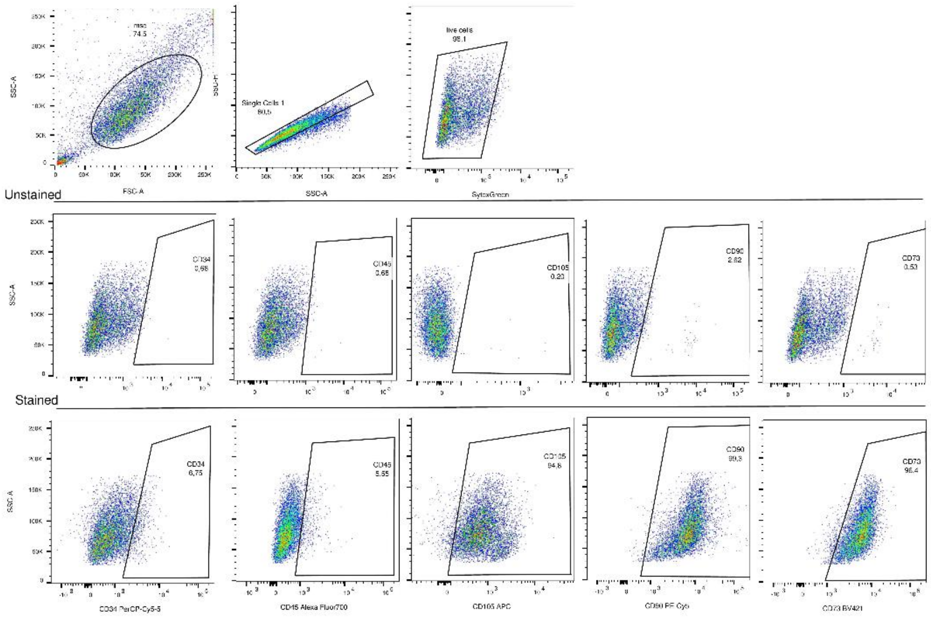

Figure S2. Flow cytometric analysis of stem cell marker expression – Donor C.

Antigen	Fluophore	Company	Cat. number
CD34	PerCP/Cy5	BioLegend, San Diego, USA	343521
CD45	Alexa Fluor700	BioLegend, San Diego, USA	368513
CD73	Brilliant Violet	BioLegend, San Diego, USA	344008
CD90	PE/Cy5	BDPharmingen, San Jose, USA	555597
CD105	APC	BioLegend, San Diego, USA	323208

Table S1. Staining reagents – antibody list.

Biological donor	% viable cells	%CD34 +cells	%CD45 +cells	%CD73 +cells	%CD90 +cells	%CD105 +cells
A	98,9	20	6,9	99,9	98,5	97,6
**B**	96,9	32,1	4,8	99,9	99,4	97,7

**C**	98,1	6,8	5,6	97,0	99,3	94,8
**D**	97,2	26,8	5,1	99,2	99,4	62,1

* For visualization, dot plots of donor C are included below

Table S2. Flow cytometry analysis of bone marrow primary cells.

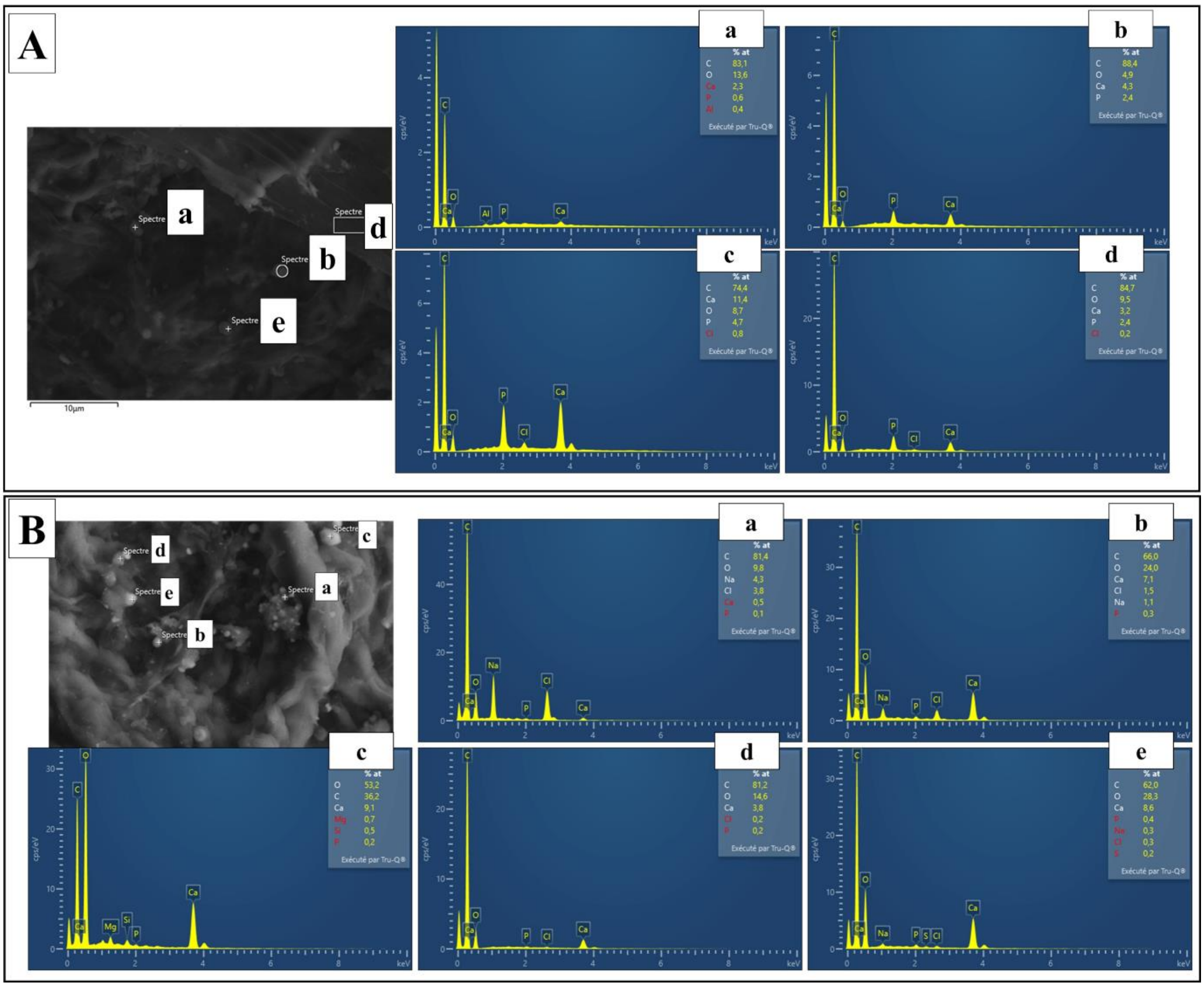

Figure S3. SEM images and EDS spectra of i) Hyb80 and ii) Zn-Hyb80 scaffolds after 7 days of incubation in SBF (As the scaffolds material is P-free, less P element index in Zn-Hyb80 is a sign of less CaP formation and inferior bioactivity).

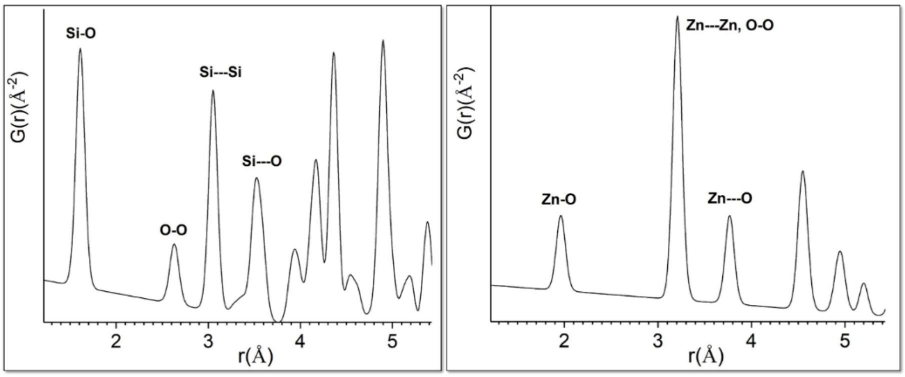

Figure S4. PDF simulations of Quartz (left: COD 1526860) and Zincite (right: COD 1011258).

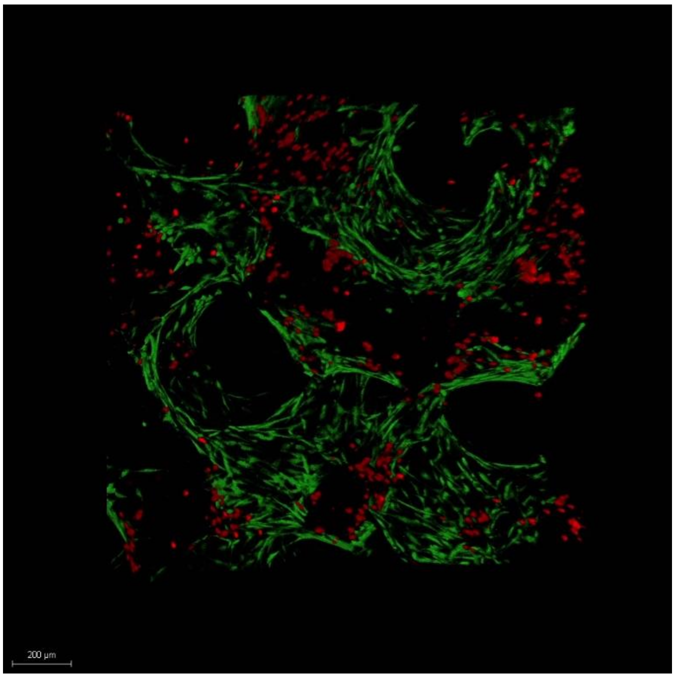

Multimedia file M1. LIVE/DEAD Staining of Hyb scaffold at 3 weeks, Scale bar is 200 µm, a link to the video should be provided in the image, the video was uploaded separately
